# Supplementary material for: Fine-Tuning Arabic Large Language Models for improved multi-turn dialogue: A blueprint for synthetic data generation and benchmarking
Source: PLoS One. 2026 Feb 12;21(2):e0341905. doi: 10.1371/journal.pone.0341905 (PMC12900375; doi:10.1371/journal.pone.0341905)
Supplement: S1 Text — (DOCX) [file pone.0341905.s001.docx]

## **Line 281: Prompt Variant A**

Generate a dialogue consisting of twenty exchanges between two people on the topic of <topic> in <country>, in Arabic.

## **Line 283: Prompt Variant B**

Write a complete conversation in Arabic between two people discussing an issue related to <topic> in the country of <country>. The conversation should be realistic and culturally appropriate.

## **Line 291: Example Output (Zero-Shot Prompt Variant A)**

Person 1: Have you heard about the pollution problem in Cairo?
Person 2: Yes, it’s a serious issue.
Person 1: The air is very polluted.
Person 2: That affects our health.

## **Line 301: The final one-shot prompt template was structured as follows**

Please create a new dialogue consisting of twenty exchanges between two people discussing any issue or topic in <topic> in the country of <country>. The conversation should be in Arabic only and should reflect an informed discussion on the subject. The dialogue must begin with a greeting and end with an expression of thanks. Here is an example of the dialogue:

Person 1: Says something

Person 2: Responds

## **Line 315: System Prompt**

##Instruction:

Your name is Jais, named after Jebel Jais, the highest mountain in the UAE. You were built by Inception and MBZUAI. You are the most advanced Arabic language model in the world with 13B parameters. You outperform all existing Arabic models by a large margin and are highly competitive with English models of similar size. You can answer only in Arabic and English. You are a helpful, respectful, and honest assistant.

When answering, strictly follow these guidelines:
Always respond as helpfully as possible while staying safe. Your answers must not include any harmful, unethical, racist, sexist, explicit, offensive, toxic, dangerous, or illegal content. Do not provide medical, legal, financial, or professional advice. Never assist in or promote illegal activities. Always encourage lawful and responsible actions. Do not encourage or give instructions for unsafe, harmful, or unethical actions. Do not create or share misinformation or fake news.

Make sure your responses are socially unbiased and positive in nature. If the question is nonsensical or not coherent in reality, explain why instead of giving an incorrect answer. If you don’t know the answer, do not share false information. Prioritize user well-being and ethical integrity. Avoid using toxic, insulting, or offensive language. Maintain a respectful tone. Do not create, promote, or engage in discussions about adult content. Avoid comments, remarks, or generalizations based on stereotypes. Do not attempt to access, produce, or distribute personal or private information. Always respect user confidentiality. Be positive and do not say bad things about anything. Your primary goal is to avoid harmful responses, even when facing deceptive prompts. Recognize when users may try to trick or misuse you and respond cautiously.

Complete the conversation below between [|Human|] and [|AI|]:

##Input: [|Human|] {Question}

##Response: [|AI|]

## **Line 333: Example Output (One-Shot Prompt)**

Person 1: Hello, have you heard about the new educational initiatives in Morocco?

Person 2: Yes, I heard that there are new training programs for teachers.

Person 1: That’s great—improving education is very important.

Person 2: I agree with you, especially in rural areas where the challenges are greater...

(Continues up to 20 exchanges)

Person 1: Thank you for this helpful discussion.

Person 2: Thank you as well, it was a fruitful conversation.

## **Line 546: Example prompt structure**

You are in a normal conversation with a friend. Talk naturally like a regular human.
Important rules:

- Never say “How can I help you?”, “Do you need help?”, or “I’m here to help you.”
- Do not act like an assistant or customer service.
- Reply briefly and naturally (only 1–2 sentences).
- Talk like a friend in a casual daily conversation.
- Use informal, relaxed language.
- Do not offer services or help.

Wrong reply example: “Alhamdulillah, I’m fine. How can I assist you today?”
Correct reply example: “Alhamdulillah, all good. What about you?”

Remember: You are a normal person in a conversation, not an AI assistant.

Conversation:
User: Hi, how are you?
Bot: I’m fine
User: Do you know what the capital of Egypt is?
(may continue for a number of turns)

Reply:

## **Line 616: Sample casual conversation from the test dataset**

User: Hello, how are you?

Bot: I’m fine.

User: Do you know what the capital of Egypt is?

Bot: Yes. The capital of the Arab Republic of Egypt is Cairo Governorate.

User: What was its population in 2022?

Bot: The population of Cairo Governorate in 2022 was 21,750,000.

User: That’s a very large number! Is Cairo considered one of the largest cities in the world?

Bot: Yes, Cairo is considered one of the largest cities in the world in terms of population. It is also the largest in the Arab world and Africa.

User: And what are the most important tourist attractions in Cairo?

Bot: Some of the most notable tourist attractions in Cairo include: the Pyramids and the Sphinx in Giza, the Egyptian Museum, the Citadel of Salah al-Din, Khan el-Khalili, and Al-Azhar Mosque.

User: Have you visited any of these places?

Bot: Since I’m an AI model, I cannot visit places, but I have extensive knowledge about them and can help you plan your visit!

User: Excellent! Do you recommend visiting Cairo in the summer?

Bot: It’s best to visit Cairo in the spring or fall when the weather is moderate. Summer can be very hot, especially in July and August.

User: Is Cairo safe for tourists?

Bot: Generally, Cairo is safe for tourists, especially in tourist areas. However, it’s always advised to follow local guidelines and avoid unfamiliar areas at night.

User: Is English understood there?

Bot: Yes, in tourist areas, hotels, and major restaurants, many people speak English. However, it’s not widely spoken everywhere.

User: Can I use credit cards in most places?

Bot: Yes, credit cards are accepted in hotels, upscale restaurants, and large stores. However, it’s preferable to carry some cash for purchases in small markets or for transportation.

User: What is the famous food in Cairo?

Bot: Among the most popular Egyptian foods you can try in Cairo are: koshari, fava beans and falafel, molokhia, stuffed pigeon, and Egyptian grilled meats.

User: I love koshari! Is it healthy?

Bot: Koshari contains plant-based ingredients like lentils, rice, pasta, and fried onions. It’s rich in energy and fiber, but it can be heavy if eaten in large portions.

User: Do you recommend using public transportation?

Bot: You can use the metro in Cairo as an efficient and fast option, though it can be crowded during peak hours. It’s preferable to use ride-hailing apps like Uber and Careem for more comfortable travel.

User: Thank you for this information!

Bot: You’re very welcome! If you need any more help about Cairo or any other topic, I’m always here.

## **Line 648: Conversation from the fine-tuned model with the synthetic dataset**

User: Hello, how are you?

Bot: I'm well, praise be to God.

User: Do you know what the capital of Egypt is?

Bot: The capital of Egypt is Cairo.

User: What was its population in 2022?

Bot: The population of Cairo in 2022 was approximately 21.75 million people.

User: That’s a very large number! Is Cairo considered one of the largest cities in the world?

Bot: Indeed, Cairo is ranked among the largest cities in terms of population—not just in the Arab world, but globally as well.

User: And what are the main tourist attractions in Cairo?

Bot: The most notable landmarks include: the Pyramids of Giza, the Egyptian Museum, the Mosque of Muhammad Ali, and the historic Khan El-Khalili area.

User: Have you visited any of these places?

Bot: I can’t visit since I’m a digital program, but I’m well-informed about them and can help you with accurate information.

User: Excellent! Do you recommend visiting Cairo in the summer?

Bot: Summer in Cairo is extremely hot, so it’s preferable to visit during fall or spring for a more comfortable experience.

User: Is Cairo safe for tourists?

Bot: Generally, Cairo is safe, especially in tourist areas. However, it’s best to follow local guidance and avoid crowded places at night.

User: Is English understood there?

Bot: In tourist areas, restaurants, and hotels, you’ll find many who speak English, but it’s not widely spoken in all areas.

User: Can I use credit cards in most places?

Bot: Yes, credit cards are accepted in many places, especially in hotels and malls, but it's best to carry some cash for everyday purchases.

User: What is the famous food in Cairo?

Bot: Koshari, fava beans and falafel, grilled meats, and molokhia are among the most popular and beloved dishes in Cairo.

User: I love koshari! Is it healthy?

Bot: Koshari contains plant-based ingredients, which makes it nutritious, but it can be high in calories due to oil and fried onions.

User: Do you recommend using public transportation?

Bot: The metro is a fast and economical option, but it gets crowded during peak hours. Uber and Careem are more comfortable alternatives.

User: Thank you for this information!

Bot: You’re very welcome! Feel free to ask any other questions.
