# Supplementary material for: Fine-Tuning Arabic Large Language Models for improved multi-turn dialogue: A blueprint for synthetic data generation and benchmarking
Source: PLoS One. 2026 Feb 12;21(2):e0341905. doi: 10.1371/journal.pone.0341905 (PMC12900375; doi:10.1371/journal.pone.0341905)
Supplement: S1 Checklist — (DOCX) [file pone.0341905.s002.docx]

**Human Participants Research Checklist**

***Complete the following if your study involved human participants or human participants’ data. These questions should be addressed for prospective and retrospective studies.***

1. Did you obtain ethics approval for this study?
   - If yes, please upload (file type “Other”) all the approval documents you received from your ethics committee to cover the entire range of the study period (i.e. the original approval document and any extension documents). Where ethics approval was obtained from more than one study location, please provide approval document(s) from all of the sites. If the original document is in another language, please also provide an English translation.

**☑ N/A**

- - If you did not obtain ethical approval, please explain why this was not required below.

The study did not involve human subjects as defined by ethical research standards. Human evaluation was conducted by two independent Arabic-speaking evaluators assessing model-generated synthetic dialogues using predefined criteria (Fluency, Relevance, Diversity on a five-point Likert scale). Evaluators participated voluntarily without compensation and conducted assessments independently. No personal data was collected, no vulnerable populations were involved, and IRB approval was not required as this constituted technical assessment of computational outputs rather than human subjects research.

1. If you prospectively recruited human participants for the study – for example, you conducted a clinical trial, distributed questionnaires, or obtained tissues, data or samples for the purposes of this study, please report in the Methods:
   1. the day, month and year of the **start and end** of the recruitment period for this study.
   2. whether participants provided informed consent, and if so, what type was obtained (for instance, written or verbal, and if verbal, how it was documented and witnessed). If your study included minors, state whether you obtained consent from parents or guardians. If the need for consent was waived by the ethics committee, please include this information.

Please state the line number(s) in the Methods where this is reported

**☑ Completed**

**Recruitment period: November 14, 2025 to December 14, 2025**

**Informed consent: The external evaluator provided consent via email. IRB approval was not required as this constituted internal quality assessment of model-generated synthetic dialogues involving no personal data collection or vulnerable populations.**

**Line numbers in manuscript where this is reported: 774-783 (full description in "Human Evaluation" sub-section under "Benchmarking and Experimental Results" section)**

1. If you are reporting a retrospective study of, for example, medical records, archived samples, survey data, please report in the Methods section:
2. the day, month and year when the data were accessed for research purposes
3. whether authors had access to information that could identify individual participants during or after data collection

Please state the line number(s) in the Methods where this is reported ______

**☑ N/A**

**No retrospective human data, such as medical records or survey data, was used. All evaluations were conducted on synthetic conversations generated by LLMs.**
