# Supplementary material for: Fine-Tuning Arabic Large Language Models for improved multi-turn dialogue: A blueprint for synthetic data generation and benchmarking
Source: PLoS One. 2026 Feb 12;21(2):e0341905. doi: 10.1371/journal.pone.0341905 (PMC12900375; doi:10.1371/journal.pone.0341905)
Supplement: Attachment — Submitted filename: Response to Reviewers.docx [file pone.0341905.s004.docx]

**Ahmed Mahmoud Misbah**
College of Computing and Information Technology
Arab Academy for Science, Technology and Maritime Transport
Cairo, Egypt
Email: a.misbah5156@student.aast.edu

**Wednesday, 01 October, 2025**

Editor-in-Chief
*PLOS ONE*

**Subject:** Response to Editorial Comments to *“Fine-Tuning Arabic Large Language Models for Improved Multi-Turn Dialogue: A Blueprint for Synthetic Data Generation and Benchmarking”*.

Dear Editor,

We thank you and the reviewers for your careful evaluation of our manuscript and for the constructive feedback provided. Below, we provide a point-by-point response to the editorial comments and detail the corresponding revisions made to the manuscript.

**1. Data availability and contact point**

*Editor comment:*

In the online submission form you indicate that your data is not available for proprietary reasons and have provided a contact point for accessing this data. Please note that your current contact point is a co-author on this manuscript. According to our Data Policy, the contact point must not be an author on the manuscript and must be an institutional contact, ideally not an individual.

*Response:*
We have revised our data availability statement. The complete dataset used in this study will now be made publicly available as part of this submission. This eliminates the need for an individual contact point and ensures that the data are accessible in line with PLOS ONE’s Data Policy.

**2. Clarification of human evaluation authorship**

*Editor comment:*

Please include a statement in your manuscript text clarifying whether the authors of this study carried out the assessment reported in the 'Human Evaluation' section.

*Response:*
We have clarified in the “Human Evaluation” subsection that the human evaluation was carried out by two of the paper’s authors.

**3. Statistical analysis**

*Editor comment:*

Has the statistical analysis been performed appropriately and rigorously? Reviewer #1: N/A

*Response:*
Assuming “N/A” refers to “Not Available,” we have strengthened the statistical analysis discussion in three places:

- In the **“Human Evaluation”** subsection, where we explain the statistical methods used,
- In **Table 9 of the “Benchmark Results”** subsection, which now summarizes the statistical outcomes of human evaluations across the four LLMs,
- In the **“Analysis of Results”** section, where we provide a detailed analysis of the findings.

**4. Additional statistical detail**

*Editor comment:*

Reporting inter-rater reliability and providing confidence intervals or statistical significance tests would enhance the rigor of the findings.

*Response:*
We have added detailed inter-rater reliability analysis for the human evaluations. This now includes percent agreement, weighted Cohen’s κ, within-one agreement, and Spearman rank correlation. These additions appear in:

- The **“Human Evaluation”** subsection,
- **Table 9** in the “Benchmark Results” subsection,
- The **“Analysis of Results”** section.

**5. Typographical and stylistic inconsistencies**

*Editor comment:*

Minor typographical and stylistic inconsistencies are present.

*Response:*
We have carefully reviewed the manuscript and corrected typographical and stylistic inconsistencies to the best of our ability.

We sincerely thank you for your guidance and for the opportunity to improve our manuscript. We hope that the revisions meet the requirements of PLOS ONE and look forward to your favorable consideration.

Warm regards,
**Ahmed Mahmoud Misbah**
(on behalf of all co-authors)
Arab Academy for Science, Technology and Maritime Transport
a.misbah5156@student.aast.edu
