# Supplementary material for: Fine-Tuning Arabic Large Language Models for improved multi-turn dialogue: A blueprint for synthetic data generation and benchmarking
Source: PLoS One. 2026 Feb 12;21(2):e0341905. doi: 10.1371/journal.pone.0341905 (PMC12900375; doi:10.1371/journal.pone.0341905)
Supplement: Attachment — Submitted filename: Response_to_Reviewers_auresp_2.docx [file pone.0341905.s005.docx]

**Ahmed Mahmoud Misbah**
College of Computing and Information Technology
Arab Academy for Science, Technology and Maritime Transport
Cairo, Egypt
Email: a.misbah5156@student.aast.edu

**Thursday, 18 December 2025**

Editor-in-Chief
*PLOS ONE*

**Subject:** Response to Editorial Comments to *“Fine-Tuning Arabic Large Language Models for Improved Multi-Turn Dialogue: A Blueprint for Synthetic Data Generation and Benchmarking”*.

Dear Editor,

We thank you and the reviewers for your careful evaluation of our manuscript and for the constructive feedback provided. Below, we provide a point-by-point response to the editorial comments and detail the corresponding revisions made to the manuscript.

**1. Reference list review**

*Editor comment:*

Please review your reference list to ensure that it is complete and correct. If you have cited papers that have been retracted, please include the rationale for doing so in the manuscript text, or remove these references and replace them with relevant current references. Any changes to the reference list should be mentioned in the rebuttal letter that accompanies your revised manuscript. If you need to cite a retracted article, indicate the article's retracted status in the References list and also include a citation and full reference for the retraction notice.

*Response:*

We have conducted a comprehensive review of the reference list and implemented the following changes:

**References removed:**

Reference 25 (Mozannar et al., 2019, "Neural Arabic Question Answering") has been removed as it was identified as a duplicate of Reference 10. All in-text citations previously referencing 25 have been updated to cite Reference 10, and subsequent reference numbers have been decremented accordingly.

Reference 46 (Singh et al., 2024, "Aya Dataset") has been removed as it was identified as a duplicate of Reference 33. All in-text citations previously referencing 46 have been updated to cite Reference 33, and subsequent reference numbers have been decremented accordingly.

As a consequence of these removals, all references following Reference 25 have been decremented by one, and all references following Reference 46 have been decremented by an additional one (i.e., decremented by two in total).

**References modified:**

URLs have been added or corrected for the following references: 8, 10, 15, 21, 22, 23, 24, 25, 26, 31, 34, 36, 37, 50, 51, and 52.

Reference text has been revised for accuracy and completeness in the following references: 12, 24, 31, 32, 33, 36, 52, 53, 54, 55, and 56.

**Reference added:**

Reference 57 (Reimers & Gurevych, 2019, "Sentence-BERT: Sentence Embeddings using Siamese BERT-Networks") has been added to provide a proper citation for the paraphrase-multilingual-MiniLM-L12-v2 sentence transformer employed in multiple phases of the study's methodology.

**2. Dataset size arithmetic**

*Editor comment:*

Dataset size math. The manuscript says there are 43,316 conversations with "precisely 20 exchanges" each, and elsewhere mentions "57 million utterances." Those numbers don't line up. Even if "exchange" means a user–bot pair (i.e., 40 turns), the total utterances are nowhere near 57M. Please correct the count everywhere and add a one-sentence derivation so a reviewer can follow the arithmetic.

*Response:*

We thank the editor for identifying this discrepancy. Upon review, we discovered computational errors in the scripts used to calculate these statistics. The scripts have been corrected, and all dataset size figures throughout the manuscript have been revised to reflect accurate values. A clear derivation of the arithmetic has been included to ensure transparency and verifiability.

**3. Data availability**

*Editor comment:*

Data availability. The Data Availability Statement points to a Google Drive folder. PLOS strongly prefers stable, citable hosting. Deposit the dataset (and scripts if possible) in a DOI-issuing repository such as Zenodo or OSF, cite the DOI in the DAS, and remove the ad-hoc cloud link. Version the release (e.g., v1.0), add a license, and include checksums.

*Response:*

In accordance with the editor's recommendation, the dataset has been deposited in Zenodo, a DOI-issuing repository that provides stable, citable hosting. The dataset is now publicly accessible at:

<https://doi.org/10.5281/zenodo.17855012>

The deposit includes version numbering, an appropriate open-access license, and checksums for data integrity verification. The Data Availability Statement in the manuscript has been updated accordingly, and the previous Google Drive link has been removed.

**4. Evaluation leakage risk**

*Editor comment:*

Evaluation leakage risk. Your benchmark is a 20% subset of the same synthetic corpus used for training/fine-tuning. That invites style overlap and optimistic scores. Clarify how you prevented duplicates (hashing, embedding similarity, seed control), and say explicitly whether the test split was frozen before model selection. If you can, add a small out-of-distribution test (e.g., held-out topics or prompts generated with different seeds) and report ID vs. OOD results.

*Response:*

We appreciate this important methodological concern. A new subsection entitled "Post Data Generation Processing" has been added to the “Methods” section, providing a comprehensive description of the procedures implemented to mitigate evaluation leakage risks. These procedures include duplicate detection mechanisms (hashing and embedding similarity checks), seed control protocols, and confirmation that the test split was frozen prior to model selection.

Additionally, our methodology included out-of-distribution (OOD) evaluation through a held-out test set comprising samples from countries and topics not represented in the training data. The revised manuscript now provides clearer documentation of these procedures, with ID versus OOD results presented in Table 14 within the "Benchmark Results" subsection and corresponding discussion in the "Analysis of Results" section.

**5. RAVEN metric specification**

*Editor comment:*

RAVEN details. Lock down the metric. Name the sentence-embedding model (exact model and version), state any text normalization (diacritics, punctuation), explain the scaling from cosine similarity to the reported "RAVEN (scaled)," and say how you aggregate from turn-level to conversation-level. Using an embedding model you also fine-tuned can bias the metric-pick a fixed external Arabic/multilingual embedder for all systems and document it.

*Response:*

The RAVEN subsection has been substantially expanded to address these concerns. The revised text now specifies: (1) the exact sentence-embedding model and version employed (a fixed external multilingual embedder not subject to fine-tuning in this study); (2) text normalization procedures applied to diacritics and punctuation; (3) the mathematical transformation from raw cosine similarity to the scaled RAVEN score; and (4) the aggregation methodology from turn-level to conversation-level metrics. These clarifications ensure full reproducibility and eliminate potential metric bias.

**6. Perplexity comparability**

*Editor comment:*

Perplexity comparability. Perplexity depends on the tokenizer and isn't apples-to-apples across models with different vocabularies. Keep PP for within-model tracking, but add a short caveat and, if you can, report a tokenizer-agnostic figure (bits-per-byte) or compute PP with a common reference tokenizer for cross-model comparisons. Also name the exact tokenizers used.

*Response:*

We have revised the "Benchmarking and Experimental Results" section to address this concern. The "Evaluation Metrics" subsection now explicitly categorizes the metrics into two groups:

1. **Within-Model Metrics:** Perplexity, used for tracking within-model performance and consistency across training and test datasets.
2. **Cross-Model Metrics:** RAVEN and human evaluation, which provide comparable assessments across different model architectures.

A new subsection on "Tokenizer Specification and Cross-LLM Comparability" has been added, which enumerates the exact tokenizers used by each model and includes an appropriate caveat regarding the limitations of cross-model perplexity comparisons. All subsequent sections have been updated to reflect this categorization.

Regarding the suggestion to report bits-per-byte or compute perplexity with a common reference tokenizer: due to time constraints and the need to prioritize other substantive revisions requested by the editor, we were unable to implement this additional analysis in the current revision. This limitation is acknowledged in the manuscript.

**7. Human evaluation reliability**

*Editor comment:*

Human evaluation reliability. Right now the raters are authors, and some weighted kappa values are near zero (even slightly negative). That weakens the claim. Bring in at least two independent raters blind to model identity, run a short calibration pass, and then report per-criterion agreement with confidence intervals. Keep the current results, but frame the author-only phase as a limitation.

*Response:*

We have addressed this concern by replacing the results from Evaluator 2 (a co-author) with results from an external evaluator. Complete details of this revised evaluation are provided in the "Human Evaluation" subsection under "Benchmarking and Experimental Results." Per-criterion agreement statistics with 95% confidence intervals are now reported. All related sections have been updated accordingly, and the "Conclusion" section includes recommendations for refining human evaluation methodologies in future research.

**8. Ethics statement**

*Editor comment:*

Ethics note. Add a one-paragraph ethics statement: internal quality assessment, no personal data collected, no vulnerable populations, no compensation, and institutional review not required under journal policy. That will stop any back-and-forth at production.

*Response:*

An ethics statement has been added to the "Human Evaluation" subsection within the "Benchmarking and Experimental Results" section. The statement confirms that the evaluation constituted an internal quality assessment, no personal data were collected, no vulnerable populations were involved, no compensation was provided to evaluators, and institutional ethics review was not required under the journal's policy.

**9. Decoding setup clarity**

*Editor comment:*

Decoding setup clarity. The generation table mixes sampling and beam search (do_sample=True with num_beams=2). If you really used beam sampling, say so; otherwise separate the setups for clarity. Fix the parameter typo repetition_penalty (it's misspelled in one place). State seeds, library versions, and whether decoding parameters were identical across models.

*Response:*

The "Prompt Engineering and Synthetic Data Generation" subsection in the “Methods” section has been revised to clarify this point. We confirm that beam-search multinomial sampling was intentionally employed, combining sampling with beam search as a deliberate decoding strategy. This is now explicitly stated in the manuscript. The typographical error in Table 2 (repetition_penalty) has been corrected. Additionally, random seeds, library versions, and confirmation that decoding parameters were consistent across all models have been documented.

**10. Computational resources summary**

*Editor comment:*

Compute/time in one place. You mention 14 days for data generation, 100 hours for fine-tuning, and 2.5 months for full benchmarking. Summarize this in a small table with hardware, key libraries (PyTorch/Transformers/PEFT versions), CUDA, OS, and random seeds. It instantly boosts trust.

*Response:*

We appreciate this suggestion for improving reproducibility. The following tables have been added to consolidate computational resource information:

- Table 3: Summary of Computational Resources Used in Dataset Generation and Timeline
- Table 6: Summary of Fine-tuning Configurations and Timeline
- Table 9: Summary of Computational Resources Used in Benchmark Evaluation and Timeline

Each table includes hardware specifications, library versions (PyTorch, Transformers, PEFT), CUDA version, operating system, and random seeds where applicable.

**11. Terminology standardization**

*Editor comment:*

Terminology. Replace "casual Arabic LLMs" with "causal (Arabic) language models" throughout. Standardize model names (LLaMA 2, Llama 3, GPT-4, Gemini, etc.).

*Response:*

The entire manuscript has been revised for terminological consistency. We have standardized all model names according to their official nomenclature (e.g., LLaMA 2, Llama 3, GPT-4, Gemini). We note that we have adopted "pre-trained Arabic language models" rather than "causal (Arabic) language models," as this terminology more accurately reflects the scope and focus of our study.

**12. PEFT implementation details**

*Editor comment:*

PEFT specifics. Since you reference IA3, packing, and use_liger, add one sentence per item (what it does, which library/version). Consider linking or archiving the training config files.

*Response:*

Table 6 ("Summary of Fine-tuning Configurations and Timeline") has been added to address this comment. The table includes explanatory descriptions of IA3, packing, and use_liger, along with their respective library versions. We believe the level of detail provided in the manuscript is sufficient for reproducibility; therefore, separate archiving of configuration files has not been pursued.

**13. Baseline fairness**

*Editor comment:*

Baseline fairness. Readers will ask whether LLaMA- or AceGPT-based baselines were fine-tuned on your training split or evaluated as-is. If they weren't fine-tuned, label them clearly as zero-/few-shot baselines or add a fine-tuned variant for a fairer comparison.

*Response:*

This concern has been addressed through the addition of two new subsections under "Benchmarking and Experimental Results": "Baseline Model Selection" and "Prompt Engineering and Model Configuration." These subsections explicitly clarify the evaluation conditions for each baseline model, including whether models were evaluated in zero-shot, few-shot, or fine-tuned settings, ensuring transparent and fair comparisons.

**14. Table labels and statistical notation**

*Editor comment:*

Tables/labels. If a column says "RAVEN (scaled)," state the range (e.g., 0–1). For inter-rater agreement, specify whether κ is quadratic-weighted and include 95% CIs.

*Response:*

This has been addressed throughout the revised manuscript. The "Human Evaluation" subsection and Tables 12, 13, and 14 in the "Benchmark Results" subsection now explicitly state the range for RAVEN (scaled), specify that Cohen's κ is quadratic-weighted, and include 95% confidence intervals for all inter-rater agreement statistics.

**15. Hyphenation artifacts**

*Editor comment:*

Hyphenation artifacts. Clean the soft-hyphen breaks from PDF export (mul-ti-turn, to-ken, in-stance, etc.).

*Response:*

The entire manuscript has been reviewed and all soft-hyphen artifacts resulting from PDF export have been removed.

**16. Data availability (Reviewer #2)**

*Editor comment:*

Have the authors made all data underlying the findings in their manuscript fully available? The PLOS Data policy requires authors to make all data underlying the findings described in their manuscript fully available without restriction, with rare exception. Reviewer #2: No

*Response:*

The complete dataset underlying the findings of this manuscript is now publicly available without restriction on Zenodo:

<https://doi.org/10.5281/zenodo.17855012>

**17. Non-functional data link**

*Editor comment:*

The data availability statement indicates that the underlying dataset has been deposited in a public repository; however, the provided Google Drive link was found to be non-functional at the time of review, preventing access to the data.

*Response:*

We apologize for this issue. The dataset has been migrated from Google Drive to Zenodo, which provides stable, persistent hosting. The dataset is now accessible at:

<https://doi.org/10.5281/zenodo.17855012>

**18. Language and typographical errors**

*Editor comment:*

Regarding the manuscript's language, it is written in generally intelligible scientific English, but a number of typographical errors, grammatical inconsistencies, and awkward phrasings were noted throughout the text, which should be addressed to enhance clarity and polish.

*Response:*

The entire manuscript has been subjected to thorough copyediting. Typographical errors, grammatical inconsistencies, and awkward phrasings have been identified and corrected to enhance clarity and overall readability.

**19. Summary of required revisions**

*Editor comment:*

It is therefore suggested that the manuscript requires minor revisions, primarily to rectify the broken data link and to undertake a thorough copyediting of the manuscript to correct language errors.

*Response:*

Both issues have been addressed. The data link has been replaced with a stable Zenodo DOI (https://doi.org/10.5281/zenodo.17855012), and the manuscript has undergone comprehensive copyediting. Additionally, we have addressed all other editorial comments detailed above to strengthen the methodological rigor and reproducibility of our work.

We sincerely thank you for your guidance and for the opportunity to improve our manuscript. We hope that the revisions meet the requirements of PLOS ONE and look forward to your favorable consideration.

Warm regards,
**Ahmed Mahmoud Misbah**
(on behalf of all co-authors)
Arab Academy for Science, Technology and Maritime Transport
a.misbah5156@student.aast.edu
